# Supplementary material for: Anxiolytic-like Effect of Inhaled Cinnamon Essential Oil and Its Main Component Cinnamaldehyde in Animal Models
Source: Molecules. 2022 Nov 18;27(22):7997. doi: 10.3390/molecules27227997 (PMC9693619; doi:10.3390/molecules27227997)
Supplement: Supplementary file 1 [file molecules-27-07997-s001.zip › molecules-2018814-supplementary.pdf]

Table S1. Downregulated genes in the hippocampus between CON and CIEO groups.

Gene symbol	Gene name	Fold change (vs. CON)	P-value
Asb4	Ankyrin repeat and SOCS box-containing 4	-1.63 ± 0.32	0.0030
Col25a1	Collagen, type XXV, alpha 1	-1.38 ± 0.17	0.0177
Dcc	Deleted in colorectal carcinoma	-1.58 ± 0.22	0.0492
Depp1	DEPP1 autophagy regulator	-1.60 ± 0.48	0.0171
Egr2	Early growth response 2	-2.09 ± 0.23	0.0075
Egr4	Early growth response 4	-1.58 ± 0.50	0.0082
Fos	FBJ osteosarcoma oncogene	-1.53 ± 0.36	0.0255
Gbp4	Guanylate binding protein 4	-1.77 ± 0.54	0.0232
Gm4971	Smad nuclear interacting protein 1 pseudogene	-1.56 ± 0.32	0.0069
Gsdma2	Gasdermin A2	-1.43 ± 0.16	0.0344
Hsf5	Heat shock transcription factor family member 5	-1.40 ± 0.13	0.0243
Insc	INSC spindle orientation adaptor protein	-1.47 ± 0.34	0.0154
Itga1	integrin alpha 1	-1.42 ± 0.13	0.0378
Nckap5	NCK-associated protein 5	-1.52 ± 0.47	0.0076
Rbm3	RNA binding motif (RNP1, RRM) protein 3	-1.46 ± 0.26	0.0440
Ssh1	Slingshot protein phosphatase 1	-1.42 ± 0.13	0.0093
Stag3	Stromal antigen 3	-1.49 ± 0.39	0.0019

Table S2. Upregulated genes in the hippocampus between CON and CIEO groups.

Gene symbol	Gene name	Fold change (vs. CON)	P-value
A930015D03Rik	RIKEN cDNA A930015D03 gene	1.65 ± 0.32	0.0207
Cytl1	Cytokine-like 1	1.89 ± 0.28	0.0007
Elmo1	Engulfment and cell motility 1	2.34 ± 0.63	0.0040
Galnt15	Polypeptide N-acetylgalactosaminyltransferase 15	1.59 ± 0.20	0.0000
Hif3a	Hypoxia inducible factor 3, alpha subunit	1.71 ± 0.15	0.0058
I830077J02Rik	RIKEN cDNA I830077J02 gene	1.73 ± 0.08	0.0030
Plin4	Perilipin 4	2.96 ± 0.29	0.0108
Rxfp1	Relaxin/insulin-like family peptide receptor 1	1.76 ± 0.13	0.0359
Sult1a1	Sulfotransferase family 1A, phenol-preferring, member 1	1.62 ± 0.13	0.0011
Tmem52	Transmembrane protein 52	1.81 ± 0.17	0.0479
Trim41	Tripartite motif-containing 41	1.70 ± 0.31	0.0191
Tshz2	Teashirt zinc finger family member 2	1.59 ± 0.26	0.0050
Uvssa	UV stimulated scaffold protein A	1.65 ± 0.29	0.0413
Vwa3a	Von Willebrand factor A domain containing 3A	1.66 ± 0.38	0.0017
Zfp868	Zinc finger protein 868	1.74 ± 0.27	0.0030

Table S3. GO Biological Process enrichment of DEGs.

GO Biological Process	Related genes
regulation of cellular protein metabolic process (GO:0032268)	*Rbm3, Ssh1*
protein modification by small protein conjugation (GO:0032446)	*Egr2, Hif3a, Trim41, Uvssa*
negative regulation of collateral sprouting (GO:0048671)	*Dcc*
Schwann cell differentiation (GO:0014037)	*Egr2*
regulation of neuron death (GO:1901214)	*Dcc, Fos*
protein ubiquitination (GO:0016567)	*Asb4, Hif3a, Trim41, Uvssa*
ethanol catabolic process (GO:0006068)	*Sult1a1*
amine metabolic process (GO:0009308)	*Sult1a1*
regulation of collateral sprouting (GO:0048670)	*Dcc*
cellular response to muramyl dipeptide (GO:0071225)	*Trim41*
cellular response to oxygen-containing compound (GO:1901701)	*Fos, Ssh1, Trim41*
positive regulation of vasculogenesis (GO:2001214)	*Asb4*
primary alcohol catabolic process (GO:0034310)	*Sult1a1*
apical protein localization (GO:0045176)	*Insc*
skeletal muscle cell differentiation (GO:0035914)	*Egr2*
positive regulation of myelination (GO:0031643)	*Egr2*
supramolecular fiber organization (GO:0097435)	*Col25a1, Elmo1, Nckap5*
response to muramyl dipeptide (GO:0032495)	*Trim41*
defense response to protozoan (GO:0042832)	*Gbp4*
regulation of vasculogenesis (GO:2001212)	*Asb4*

Table S4. WikiPathway enrichment of DEGs.

WikiPathway	Related genes
Signaling of Hepatocyte Growth Factor Receptor WP313	*Itga1, Fos*
Chromosomal and microsatellite instability in colorectal cancer WP4216	*Dcc, Fos*
Arylamine metabolism WP694	*Sult1a1*
Neuroinflammation WP4919	*Fos*
Serotonin and anxiety-related events WP3944	*Fos*
Transcriptional cascade regulating adipogenesis WP4211	*Egr2*
Brain-derived neurotrophic factor (BDNF) signaling pathway WP2380	*Egr2, Fos*
Regulation of Actin Cytoskeleton WP51	*Itga1, Ssh1*
Hepatitis B infection WP4666	*Egr2, Fos*
MAPK pathway in congenital thyroid cancer WP4928	*Fos*
Myometrial relaxation and contraction pathways WP289	*Fos, Rxfp1*
NOTCH1 regulation of endothelial cell calcification WP3413	*Itga1*
Serotonin and anxiety WP3947	*Fos*
Sulfation Biotransformation Reaction WP692	*Sult1a1*
Estrogen metabolism WP697	*Sult1a1*
Galanin receptor pathway WP4970	*Sult1a1*
Tamoxifen metabolism WP691	*Fos*
miRNA targets in ECM and membrane receptors WP2911	*Itga1*
Estrogen signaling pathway WP712	*Fos*
Photodynamic therapy-induced NFE2L2 (NRF2) survival signaling WP3612	*Fos*
